# The Application of Biomedical Engineering Techniques to the Diagnosis and Management of Tropical Diseases: A Review

**DOI:** 10.3390/s150306947

**Published:** 2015-03-23

**Authors:** Fatimah Ibrahim, Tzer Hwai Gilbert Thio, Tarig Faisal, Michael Neuman

**Affiliations:** 1 Department of Biomedical Engineering, Faculty of Engineering, University of Malaya, 50603 Kuala Lumpur, Malaysia; E-Mails: gilbert_thio@hotmail.com (T.H.G.T.); tarig_28@yahoo.com (T.F.); 2 Centre for Innovation in Medical Engineering (CIME), Faculty of Engineering, University of Malaya, 50603 Kuala Lumpur, Malaysia; 3 Faculty of Science, Technology, Engineering and Mathematics, INTI International University, 71800 Nilai, Negeri Sembilan, Malaysia; 4 Faculty-Electronics Engineering, Ruwais College, Higher Colleges of Technology, Ruwais, P.O Box 12389, UAE; 5 Department of Biomedical Engineering, Michigan Technological University, Houghton, MI 49931, USA; E-Mail: mneuman@mtu.edu

**Keywords:** biomedical engineering, tropical diseases, micro/nano-fluidic platform, artificial intelligence, bioelectrical impedance, lab-on-chip, diagnostic, dengue

## Abstract

This paper reviews a number of biomedical engineering approaches to help aid in the detection and treatment of tropical diseases such as dengue, malaria, cholera, schistosomiasis, lymphatic filariasis, ebola, leprosy, leishmaniasis, and American trypanosomiasis (Chagas). Many different forms of non-invasive approaches such as ultrasound, echocardiography and electrocardiography, bioelectrical impedance, optical detection, simplified and rapid serological tests such as lab-on-chip and micro-/nano-fluidic platforms and medical support systems such as artificial intelligence clinical support systems are discussed. The paper also reviewed the novel clinical diagnosis and management systems using artificial intelligence and bioelectrical impedance techniques for dengue clinical applications.

## 1. Introduction

The original designation of certain diseases as being tropical can be dated back to the 1898 publication of Sir Patrick Mansonʼs *Tropical Diseases: A Manual of the Diseases of Warm Climates* [1]. Tropical diseases (TD) are diseases that are widespread in or unique to the tropical and subtropical regions. TDs are prevalent in hot and humid climates. TDs are caused by pathogenic agents such as bacteria, viruses, parasites or fungi and are most often transmitted through carriers or vectors such as insects and nematodes. These insects may carry a parasite, bacterium or virus that is transmitted via their bite which disperses the infectious agent by subcutaneous blood or saliva exchange in humans and animals. Examples of tropical diseases include malaria, tetanus, hepatitis, American trypanosomiasis (chagas), dengue, yellow fever, cholera, nipah virus, and many others. We will look at some of these where biomedical engineering approaches have contributed to their diagnosis and treatment.

Rapid, efficient and inexpensive diagnosis of TDs is vital for the effective treatment and quality management of diseases in tropical regions. Diagnosis of TDs consists of a wide range of methods including serological testing of pathogenic markers such as protein, antigen, and antibody (using the enzyme-linked immunosorbent assay (ELISA) technique and various test kits), x-rays and physical examination, as well as performing bacteria and fungi culture techniques. These methods typically require a sample of bodily fluid, such as blood, sputum, or urine and/or stool samples. However, the management and diagnosis of TDs face several challenges such as prolonged turnaround time for assessment of specimens, high cost, a controlled environment, highly trained personnel, and large blood or bodily fluid samples. Associated measurements are often invasive, and in order to be useful, diagnostic methods must be accurate, simple and affordable for the population for which they are intended. They must also provide a result in time to institute effective treatment and patient isolation when necessary. Current diagnostic methods for TDs are frequently faced with challenges such as inconsistency in specificity and sensitivity, a long turnaround time to receive results, and high cost or specialization (which requires either an advanced and expensive laboratory setup, or skilled technicians and clinicians, or both).

Recent research and studies in the past decade have proposed and introduced various biomedical engineering approaches that attempt to address the issues faced in the diagnosis of tropical diseases. This review paper focuses on current biomedical engineering approaches for the more prominent diseases of dengue, malaria, cholera, schistosomiasis, lymphatic filariasis, ebola, leprosy, leishmaniasis, and American trypanosomiasis (chagas). Many of these approaches can be extended to other diseases as well.

These diseases were chosen as examples due to their severity and endemic nature and the perceived limitations of the current diagnostic methodology. In recent years outbreaks of dengue have been escalating and spreading geographically throughout the world with high mortality rates when timely treatment is not administered [2,3]. The number of infections of malaria and schistosomiasis is in the range of hundreds of millions, with the annual mortality rates in the range of hundreds of thousands [4,5]. From 2009 to 2010, the number of cases and deaths due to cholera has increased by about 50% [6], while sporadic outbreaks of ebola have been noted to be extremely deadly, killing the patient within 2 days after acquiring the infection [7]. The disease of lymphatic filariasisis is wide spread, infecting 120 million people and disfiguring 40 million worldwide [8], while leprosy has an annual infection rate of 200,000 a year, and causes permanent damage to the skin, nerves, limbs and eyes if untreated [9]. Around the world, millions of people are infected with leishmaniasis and American trypanosomiasis (chagas), and millions more are at risk due to the endemic nature of these diseases [10,11,12].

Although the diseases are different, many of the biomedical engineering approaches are common for their diagnosis and treatment. Table 1 presents the relation between the diseases and biomedical engineering approaches for their Diagnosis and Treatment. In the following sections we review biomedical engineering approaches that are currently available for diagnosis and establishing prognosis of the tropical diseases listed in Table 1. As seen in the table, a wider variety of biomedical engineering approaches have been applied to diseases such as dengue and malaria than some of the diseases on the right side of the table.

**Table 1:** Relation between the Tropical Diseases and their Diagnosis and Treatment via Biomedical Engineering approaches.

Biomedical Engineering Approach to Diagnosis and Treatment	**Tropical Diseases**								
	Dengue	Malaria	Cholera	Schistosomiasis	Lymphatic Filariasis	Ebola	Leprosy	Leishmaniasis	Chagas
Bioelectric Impedance Analysis	X		X	X					
Bioelectric Properties		X							
Biosensor						X		X	X
Clinical Decision Support Systems	X								
Dielectrophoresis		X							
Echocardiography	X								
Electrocardiography	X								
Image Processing		X							
Imaging: Computed Tomography (CT) and Magnetic Resonance Imaging (MRI)				X	X		X		
Imaging: Ultrasonic	X			X	X		X		
Laser Doppler Velocimetry	X								
Microarray chip		X							
Microfluidics and Lab-on-a-Chip	X	X	X						
Paper-based Diagnostic	X	X							
Plethysmography	X								

## 2. Dengue

One of the most rapidly spreading mosquito-borne viral diseases in the world is dengue. The incidence of dengue has increased in the last 50 years as a result of increasing geographic expansion to new countries and to rural areas [2]. Annually, the World Health Organization (WHO) estimates the occurrence of 50 million dengue infections occurring and approximately 2.5 billion people facing threats of dengue since they live in dengue endemic areas [13].

According to the WHO 1997 guidebook of Dengue Haemorrhagic Fever: Diagnosis, Treatment, Prevention and Control, 2nd ed. [13], some patients develop dengue fever (DF) in the early stage after a person is infected with the dengue virus and recover after the fever subsides, while other patients may progress on to develop dengue haemorrhagic fever (DHF).

Laboratory and clinical diagnosis are used to diagnose the dengue patient. The clinical diagnosis and severity of DHF were graded from grades I to IV based on the WHO guidebook recommendation in 1997 [13]. The WHO guidelines define Grade I as patients having a fever accompanied by nonspecific constitutional symptoms with the only haemorrhagic manifestation being a petechial rash. Grade II is defined as patients having a spontaneous bleeding from any site on their skin. Grades III and IV are known as dengue shock syndrome (DSS). Grade III is where a patient has circulatory failure manifested by rapid and weak pulse, narrowing of pulse pressure (20 mmHg or less) or hypotension, with the presence of cold clammy skin and restlessness. Grade IV (DSS) is defined as patients having profound shock [13].

With the WHO 1997 dengue severity classification guideline [13], many clinicians experienced several difficulties especially in the critical cases such as the DHF patients which may experience significant plasma leakage that may lead to haemorrhage and organ impairment. The decision to admit those patients to the hospital in order to monitor their plasma leakage is a great challenge due to the overlapping of present medical classification criteria for establishing the risk of dengue patients [14,15]. On the other hand, physicians cannot decide to admit all patients because this will have an impact on the cost and quality of health care due to the high incidence of dengue in the tropics. Thus, to overcome these difficulties and to assist clinicians in determining the severity of the infection and how patients should be treated, the WHO has improved and introduced the new [2] guidebook which defines the dengue patients’ classification according to a few levels of severity. This guideline has been approved in year 2010 and valid until year 2014. In this new guidebook, the dengue patients are no longer classified as DF and DHF, instead patients are divided into two simplified groups. They are either severe or non-severe dengue patients. The non-severe dengue patients are further classified into two subgroups: patients with warning signs and those without warning signs.

In the new WHO 2009 guidebook [2], the classification criteria for non-severe dengue without warning signs are fever and any two of the following: nausea/vomiting, rash, aches and pain, a positive tourniquet test, leucopoenia, and there may even be a combination of these warning signs. The warning signs for non-severe dengue with warning signs are: abdominal pain or tenderness, persistent vomiting, clinical fluid accumulation (pleural effusion/ascites), mucosal bleeding, lethargy, restlessness, liver enlargement (>2 cm), and an increase in hematocrit (HCT) concurrent with a rapid decrease in platelet count. Such patients with these warning signs require strict observation and medical intervention. Severe dengue infections are characterized by significant plasma leakage, severe bleeding, and severe involvement of organs such as the liver. This causes liver enzymes such as aspartate aminotransferase (AST), or the alanine aminotransferase (ALT) to be elevated with readings of ≥1000 units/L.

Laboratory diagnosis of dengue patients is used to detect the dengue virus. Currently, a definitive diagnosis of dengue infection can be made only in the laboratory, either through virus isolation, detection of viral antigen or ribonucleic acid (RNA) present in serum or other bodily fluid, detection of antibodies present in serum, or a combination of these techniques [2,16]. At the early stage of infection, virus isolation and the detection of nucleic acid or antigen is used to make a diagnosis of dengue infection; however at the end of the acute phase of infection serological methods are more suitable [2]. A number of commercially rapid test kits (or coated strips) have been developed by various companies such as Korea’s Standard Diagnostics, Biorad and Panbio. These kits are able to detect a combination of either antigen or antibodies [3].

Although rapid laboratory diagnosis is very important and highly desirable, of equal importance is the recovery rate of the patient and the patient’s quality of life after recovery. To date, there is no effective vaccine or antiviral drug for dengue [2]. Some of the dengue patients might recover spontaneously while others face critical plasma loss that can lead to fatality [13]. Serology tests are not able to diagnose the micro-vascular status (micro-vascular leakage or plasma leakage) of the patient which is one of the major pathophysiological changes during dengue infection. Nevertheless the fatality of dengue disease can be reduced by close monitoring of patients to detect the onset of plasma leakage and administer prompt intravenous fluid replacement [17].

### 2.1. Biomedical Engineering Approaches

In order to overcome some of the difficulties in conventional dengue infection diagnostic methods, a few biomedical engineering (BME) approaches were introduced by proposing non-invasive tools to diagnose and classify the disease severity. These included techniques such as ultrasound imaging, echocardiography, electrocardiography, plethysmography, laser Doppler velocimetry, bioelectrical impedance, and intelligent clinical decision support systems. However, many of these papers reviewed in the following sections were based on the WHO 1997 [13] dengue classification as the new WHO 2009 [2] classification was introduced in the year of 2010, after these papers have been published.

#### 2.1.1. Ultrasound

The critical stage in dengue occurs when the capillary permeability increases which leads to plasma leakage and therefore loss of plasma volume. The presence of pleural effusion and ascites are often used to determine the degree of plasma leakage. These are clinically detectable through physical examination techniques such as auscultatory percussion, and imaging techniques such as chest radiography, and also abdominal and thoracic ultrasonic imaging. Accordingly, several studies have used ultrasound as an aid for diagnosing dengue disease [18,19,20].

In the study by Srikiatkhachorn *et al.* [18] ultrasound has been employed to delineate the locations and the timing of plasma leakage in DHF. In the study, one hundred fifty-eight suspected dengue cases classified as DF, DHF, or other Febrile Illness (OFI) based on serology and evidence of plasma leakage including hemoconcentration and pleural effusion, were investigated. Ultrasound examinations of the abdomen and right thorax of patients were performed to detect ascites, thickened gall bladder wall, and pleural effusions. The results indicated that the timing of the plasma leakage was around the time of defervescence. The Pleural effusion was the most common ultrasonographic sign of plasma leakage while the thickening of the gallbladder wall and ascites were not associated as much in determining the plasma leakage. Significantly, plasma leakage of 12 out of 17 DHF cases who did not meet the WHO criteria for hemoconcentration signs was detected by ultrasound. The study concluded that ultrasound imaging is a useful tool for detecting plasma leakage in dengue infection.

Another study by Venkata *et al.* [19] was conducted to determine the importance of the ultrasound to clinical and laboratory profiles in diagnosing DF or DHF and to determine the usefulness of ultrasound in predicting the severity of the disease. One hundred twenty-eight suspected dengue patients (40 serologically negative for dengue fever and 88 serologically positive cases) were studied. Results of 32 patients from the 88 cases who were examined on the second to third day and repeated on fifth to seventh day showed that 100% had gall bladder wall thickening and pericholecystic fluid. Follow-up ultrasound on the fifth to seventh day showed ascites in 53%, left pleural effusion in 22%, and pericardial effusion in 28%. The results of the remaining 56 patients who were examined on the fifth to seventh day of fever for the first time showed that 100% had gall bladder wall thickening, 96% had ascites, 87.5% had right pleural effusion, and 66% had left pleural effusion. Contrary to the previous study, this study reported that thickened gall bladder wall, pleural effusion, and ascites should strongly favour the diagnosis of dengue fever.

In a separate work by Setiawan *et al.* [20], a study was conducted to examine the relationship between the clinical severity of 148 DHF patients (73 grades I and II; 75 grades III and IV) and their sonographic findings. Ultrasonography results revealed that the main features presented with grades I and II DHF patients were hepatomegaly 49%, ascites 34%, gallbladder wall thickening 32%, and pleural effusions 30%. On the other hand, the main features detected in DHF patients grades III and IV groups were pleural effusions, ascites and gallbladder wall thickening 95%, pararenal and perirenal fluid collections 77%, hepatomegaly 56%, and pancreatic gland enlargement 44%. The study concluded that ultrasound may be useful for early prediction of the severity of DHF.

#### 2.1.2. Echocardiography and Electrocardiography (ECG)

Echocardiography and electrocardiography (ECG) were utilized in several studies to assess cardiac function of dengue patients [21,22,23].

Acute shock in severe DHF cases may occur in parallel with accumulation of fluid in serous body spaces such as the pleural, peritoneal, and pericardial cavities [21]. Pelupessy *et al.* [21] investigated the implementation of echocardiography in diagnosing dengue patients since echocardiography is a very sensitive method for detecting any small quantity of pericardial effusion The study showed that, although no signs of pericardial effusion could be determined on physical examination of DHF patients associated with severe shock and through ECG and radiological procedures, echocardiogram results were able to clearly show a small amount of fluid. Thus this technique is only recommended for the application of acute shock dengue patients.

In 1998, Wali *et al.* [22] employed radionuclide ventriculography, echocardiography, and ECG to assess cardiac function of 17 DHF and DSS patients. The radionuclide ventriculography results revealed that, the mean left-ventricular ejection fraction was 41.69%. Seven patients had an ejection fraction of less than 40%. Global hypokinesia was detected in 70.59% of the patients. The echocardiography results showed that the mean ejection fraction was 47.06%. The mean ejection fraction of the 8 DSS patients was 39.63%. Five (67.5%) of those patients had an ejection fraction below 40%. Radionuclide ventriculography and echocardiography showed no abnormalities after 3 weeks of follow up for five patients who had ST and T changes in their electrocardiogram. The ejection fraction was more than 50% in these cases. Within 3 weeks, the Global hypokinesia also improved and ECG changes reverted back to normal. The study concluded that acute reversible cardiac insult may be noticed in DHF/DSS and could be responsible for hypotension/shock seen in some of these patients. It was recommended that further studies are carried out to establish the pathogenic mechanisms of cardiac dysfunction in patients with DHF and DSS.

In 1993, Yusoff *et al.* [23] performed echocardiograms and ECGs on 28 consecutive adult patients with a clinical diagnosis of dengue infection. Twenty-three dengue patients were serologically confirmed (22 DHF grades I and II; 1 DF). 87% of the serologically confirmed dengue patients had abnormal ECGs and/or echocardiograms. Of these, 65% had abnormal ECGs that consisted of conduction abnormalities, ST segment elevation, T wave inversion, and sinus bradycardia. Fifty-two percent had abnormal echocardiograms which showed pericardial effusion, abnormal systolic and diastolic functions, left ventricular dilatation, and tricuspid regurgitation. The authors declared that ECG and echocardiographic abnormalities are common during the acute phase of DHF. They recommended early detection of cardiac involvement as a way of identifying the more severe forms of dengue so that appropriate treatment can be initiated as early as possible.

#### 2.1.3. Strain Gauge Plethysmography

Liquid metal (Mercury-in-silicon elastomer tube) strain gauge plethysmography has been used in various studies to assess the microvascular permeability in dengue patients [24,25]. Gamble *et al.* [24] investigated the possible use of age-related changes in microvascular permeability as a health indicator, and it was found that the value was highest in DSS young children. These findings indicated that children significant factor in the susceptibility of children to DSS using strain gauge plethysmography [24]. Both adults and children DSS patients were found to have higher vascular permeability than the healthy control data, and the value was highest in the young children. These findings indicated that children are more susceptible to develop hypovolaemic shock than adults in DHF and other conditions characterized by increased microvascular permeability.

Bethell *et al.* [25], on the other hand, investigated whether the underlying pathophysiology of DSS is distinct from the milder forms of the disease by assessing the microvascular permeability also using strain gauge plethysmography. Three groups were investigated: children with DSS, DHF without shock, and in healthy children. The mean coefficient based on the statistical analysis of microvascular permeability for the patients with dengue was 50% higher than in healthy control patients. However, there was no significant difference in the permeability between the two groups of patients with dengue, which suggests the same underlying pathophysiology. This study also demonstrated that increased microvascular leakage occurs in children with DHF, and is most pronounced in children with DSS. However, the time taken to conduct the measurement was long (40 min average), and the procedure of cuffing the patients may put the patients at risk by inducing more capillary leakage.

#### 2.1.4. Laser Doppler Velocimetry

Laser Doppler velocimetry has been widely used for assessment of various physiological parameters, particularly involving blood perfusion and circulation [26,27,28,29]. The technique has also been recently applied to DHF patients to evaluate the microcirculation changes due to plasma leakage and increase of microvascular permeability [30]. The preliminary findings of this study indicated that there were significant differences between basal laser Doppler flux in normal healthy subjects and DHF patients. These results also implied that the technique has the potential as an indicator to microcirculatory changes in DHF patients. The study has not; however been able to conclusively differentiate the DHF severity stages using this technique.

#### 2.1.5. Bioelectrical Impedance

Over the years, bioelectrical impedance analysis (BIA) has demonstrated its utility as a non-invasive method for measurement and diagnosis in several medical applications. BIA evaluates the human body composition such as mass distribution (*i.e.*, body cell mass and extracellular mass), and water compartments (*i.e.*, intracellular water, extracellular water) by passing a small current through it [31]. The majority of the impedance measurements use four electrodes to minimize electrode-skin impedance effects. A small high-frequency current (50 kHz) is passed between two of the electrodes while the voltage drop across the same area is then measured using the other pair of electrodes. The ratio of the voltage drop to the current determines the resistance and reactance (Xc) of the body segment being measured. This data can then estimate the extracellular water (ECW), intracellular water (ICW), fat free mass (FFM), and fat mass through recognized equations [32].

BIA and bioelectrical impedance spectroscopy (BIS) have been described as a potentially reliable method to assess clinically significant changes in extracellular and total body water in dengue patients [33,34,35,36,37]. It could be a useful tool as a proxy for formal dilution methods to assess fluid shifts. Pierson and Wang (1986) have also proposed that an elevation of the extracellular water (ECW)/intracellular water (ICW) ratio in dengue patients, determined by dilution techniques, is a sensitive but nonspecific marker for the presence of systemic disease, and total body impedance measurements are especially useful in this determination.

In dengue infections several approaches were followed for utilizing the BIA technique. The hydrational profile which reflects the distribution of body water between the intra and extracellular space in dengue patients was investigated [38,39,40,41,42,43,44].

Studies conducted by Klassen *et al.* [42], Ibrahim *et al.* [38], and Mazariegos *et al.* [44] have shown that BIA was sensitive in determining the hydrational profile in dengue patients. Klassen *et al.* [42] studied the changes in hydrational status during the acute phase of classical dengue fever in 9 adult patients. They studied the effects of the acute classical dengue fever on ECW, ICW, and total body water (TBW) by comparing conventional dilution techniques with the outcome variables from whole body impedance spectroscopy (BIS), extracellular fluid resistance (R_ecf_), and intracellular fluid resistance (R_icf_) [42]. Two groups were investigated: a reference group comprised of 15 subjects without acute or chronic illness and dengue patients. The dengue patients were investigated on admission with febrile presentation (DI), at discharge after the defervescence of the fever cycle at about five days post-admission (DII), and seven days thereafter (DIII). The results revealed that the total body water did not change during the course of the disease and was not different from that in normal healthy subjects. However, the ratios of ECW/TBW and ECW/ICW reflected that body water shifted from the intracellular to the extracellular compartment in patients from the acute phase to convalescence. This ratio was also higher in convalescent dengue patients (DIII) compared to the reference group. The results also showed an association between increasing ECW, from the acute phase of the disease to convalescence and decreasing the R_ecf_ and the R_ecf_/R_icf_ ratio. Moreover, the R_ecf_ and R_ecf_/R_icf_ values were higher in the acute phase (DI) of dengue fever compared to those of the non-dengue subjects. The study concluded that relative expansion of ECW during the course of the disease and in the convalescence stage as determined by measuring body impedance can be used to monitor the dengue fever progression.

Fang *et al.* [45] reported a biosensor platform for dengue fever detection from patient serum. Their sensor was based on a non-faradic process where an integrated sensor captures the dengue antibody selectively from the sample. A thin film of sol-gel derived barium strontium titanate (BST) was coated on the immunosensor surface, and then the surface was modified with an organic self-assembled monolayer (SAM). In addition, pre-inactivated dengue virus was indirectly immobilized on the surface to act as a sensing probe to capture the dengue antibody. Finally, the modified surface was based on a finger shaped electrode where the output impedance/current will change in correlation with the presence and concentrations of dengue antibody in the serum sample. The work was conducted at frequency ranges of 0.1 Hz to 1 MHz.

**Figure 1:**
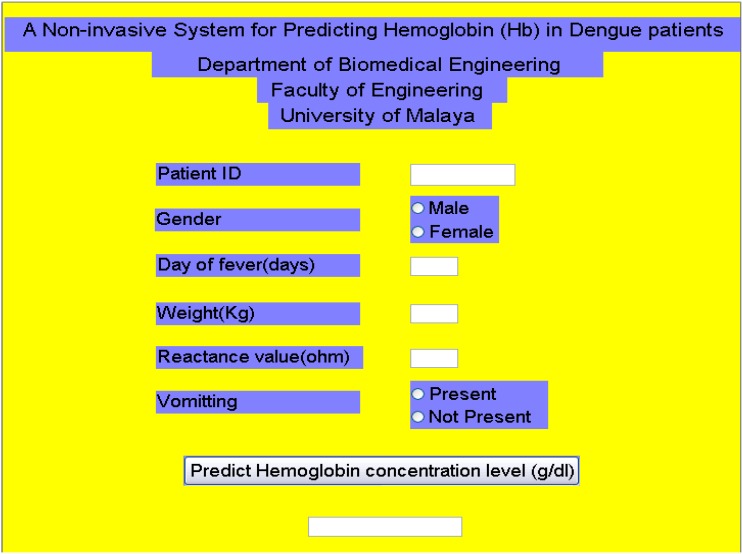
Non-invasive haemoglobin modelling of dengue patients using bioelectric impedance analysis and artificial neural networks.

Ibrahim *et al.* [46] monitored and modelled the *hemoglobin* (Hb) status in dengue patients using the BIA parameters. The Hb status was used since it is directly related to the Hct status which can be used to determine the degree of microvascular permeability in dengue patients. The BIA was employed to construct a model for predicting Hb in dengue patients using the multivariate analysis technique. Eighty-three (47 males and 36 females) serologically confirmed DF and DHF patients were studied during their hospitalization. The data consisted of all the investigated parameters in BIA, patientsʼ symptoms, and demographic data. Four predictors: reactance, gender, weight, and vomiting were found to be the most significant parameters for predicting the Hb levels in dengue patients. The study concluded that the single frequency bio-impedance technique and Multiple-linear-regression analysis is insufficient to monitor Hb for dengue patients since this analysis only explains approximately 42% of the Hb variation. This model has been enhanced by utilizing a non-linear artificial neural network (ANN) and achieved 74% accuracy [47]. The graphical user interface for the model is shown in Figure 1. The enhanced model is able to predict the Hb concentration which helps to determine the degree of microvascular permeability in dengue patients based on the above mentioned data.

Additional enhancement of the model was achieved by employing the nonlinear autoregressive moving average with exogenous input (NARMAX) approach [48]. Three different NARMAX model order selection criteria, namely FPE, AIC, and Lipschitz were evaluated. The results gave 88.40% prediction accuracy by using the Lipschitz number approach. The study concluded that the NARMAX model yields better accuracy compared to the autoregressive moving average with exogenous input (ARMAX) model which achieved 76.70% accuracy.

#### 2.1.6. Dengue Clinical Decision Support Systems

A physician’s knowledge would be sufficient to diagnose diseases that are directly related or corroborate to the encyclopaedic aspects of medicine. However, the complexity of diseases such as dengue and the many overlapping levels of its severity has created many difficulties for the physician to predict the disease prognosis. Accordingly, there is a crucial need for a decision support system to assist the health care provider in understanding the disease and to plan for its treatment. This is especially true when providers with less training than a physician are caring for patients, a factor crucially important in the developing world. In dengue, several approaches have been followed to achieve this goal including the self-organized map, multilayer feed-forward neural networks (MLFFNN), and adaptive neuro-fuzzy inference system (ANFIS) techniques.

##### Self-Organizing Map (SOM)

The self-organizing map (SOM) is an unsupervised neural network that is considered as one of the most powerful aids for visualizing, analyzing, and understanding the complexity of high-dimensional data. It receives a number of different multivariable input samples, discovers significant relationships in these samples, and presents them into a two dimensional map. This map contains different data clusters (prototypes), each of them consisting of samples that have similar features. Similarly, relationships within the data and cluster structures can be visualized and interpreted. Therefore, the SOM can be considered as an exploratory data analysis tool for generating hypotheses on the relationships among the data. The SOM has been widely used in medical applications [49,50,51,52,53]. Typical SOM can be visualized by using the U-matrix and the component planes. The U-matrix visualizes the distances between map units which is used to show the cluster structure of the map. Normally it is colored and these colors represent the distance between map units [54]. Colors with high values in the U-matrix indicate a cluster border while colors with low value indicate the clusters themselves. The value of the color is presented in the color scale beside the U-matrix. On the other hand, the component planes present values of all variable in each U-matrix map unit.

**Figure 2:**
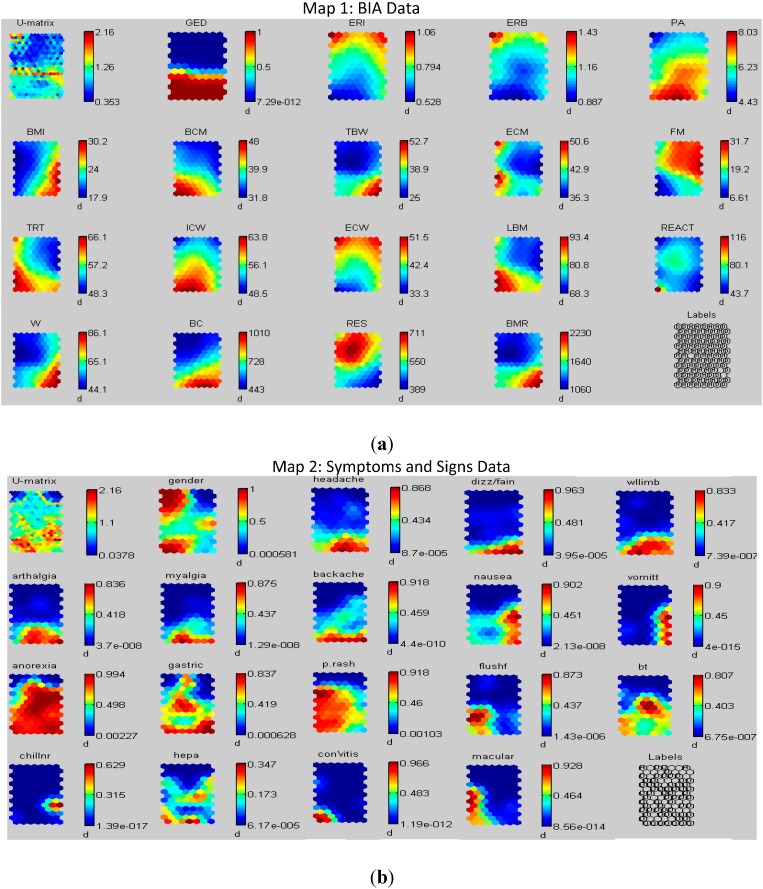
(**a**) Visualization of the self organizing maps for the bioelectric impedance analysis parameters; (**b**) Symptoms, and signs data. Reproduced with permission [47].

In dengue infections, the SOM was employed to identify the non-invasive significant prognosis factors that can distinguish between dengue patients and healthy subjects and also distinguish between the male and female patients [47]. The study presented a new approach to determine the significant prognosis factors in dengue patients utilizing the SOM. This technique showed the significant factors that can differentiate between dengue patients and the healthy subjects. Three hundred twenty-nine samples (210 dengue patients and 119 healthy subjects) were used in the study. Each sample contained 35 predictors (17 BIA parameters, 18 symptoms/signs). Two SOMs were constructed as shown in Figure 2. Each map contains the U-matrix (on the top left of the map) and the component planes that represent the value of the variable in the U-matrix. The bottom left of each map shows the labels which indicate the type of patient in each cluster of the U-matrix map (healthy subject (H) or dengue patients (D)).The first map was constructed based on the BIA parameters data (variables) while the second map utilized the symptoms and signs data (variables). By visualizing the U-matrix and investigating the similarity between the clusters in the U-matrix and the component planes, the correlations between the dengue patients and the prognosis factors form the symptoms and signs and BIA parameters can be defined. The results revealed that, the significant BIA prognosis factors for differentiating the dengue patients from the healthy subjects were reactance, ICW, ratio of ECW/ICW, and ratio of the extracellular mass to body cell mass. On the other hand, abdominal epigastic pain, petechiea rash, and bleeding tendency were the main signs and symptoms that were present in dengue patients.

Due to the limitations of the WHO 1997 classification criteria that have been used to classify the severity of dengue patients, Faisal *et al.* [55] employed clustering, the SOM’s technique to determine new criteria that may help classify dengue patients based on disease severity. This technique aims to apply the K-mean clustering technique to cluster the SOMʼs prototypes rather than clustering the data directly to enhance the data clustering. Generally, the implementation of this approach is performed in two stages: First, the SOM is trained to identify the prototypes of the dengue patients’ data, and second, the K-mean clustering technique is implemented to cluster those prototypes. As a result, three criteria were then defined to classify the level of risk in dengue patients. The results were validated by comparing them to some other dengue researchers’ findings as well as the WHO criteria [2,13]. By using this technique, important results were obtained: the new risk criteria classified 33% of the DF patients as high risk dengue patients. Those patients might not be hospitalized according to the WHO criteria since they were classified as DF patients. However, those patients were classified as high risk by using the new criteria and thereby they might be at risk and face death if they are not closely monitored to detect the onset of plasma leakage. Another significant result was that 65.5% and 57.7% of the patients who were classified by WHO as DHF I and DHF II, respectively, were classified by the new criteria as low risk dengue patients. Those patients need not be hospitalized since they are classified as low risk patients and therefore the savings on the cost of the hospital admissions can be substantial. This result agrees with other researchers’ findings [14] and the recent WHO guidelines which indicates there is a problem using the existing WHO classification due to the changes in the epidemiology of dengue, and there is a high potential for the clinicians’ decision to be based the levels of severity for classifying the patients [2].

##### Multilayer Feed-Forward Neural Networks (MLFFNN)

ANNs have been successfully applied to several problems in dengue disease. Ibrahim *et al.* [56] employed the multi-layer perceptron (MLP) network trained via the back-propagation (BP) algorithm to develop a prediction system for predicting the day of fever reduction in dengue patients due to the fact that the progression of the DHF patient to DSS occurs following this day. Ninety percent prediction accuracy was achieved by using this approach. The study concluded that since most of the dengue patients were sick during or around the time of fever reduction, this ANN might be very promising to assist clinicians in the early determination of prognosis and in prescribing the management plan for their patients.

**Figure 3:**
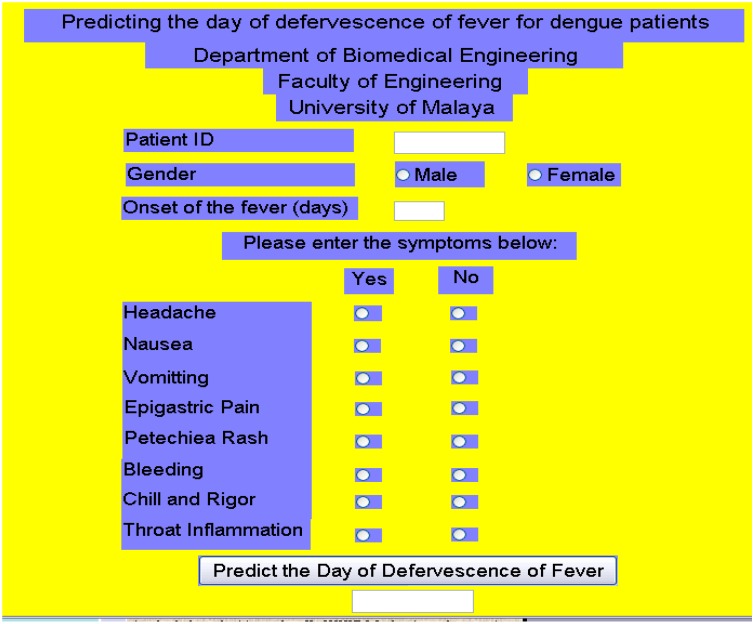
Graphic interface screen for the prediction of day of defervescene of fever in dengue patients.

The network architecture designed by Ibrahim *et al.* [56] has been used to develop a GUI as shown in Figure 3. The user interface is comprised of a few dialog boxes and radio buttons that request patient information and input data for the prediction process. The patient information includes patient identification and gender. The radio buttons are used to simplify user selection of the symptoms and the signs presented by the dengue patients. The “predict the day of defervescence of fever” button gives the estimated day of fever reduction. The entire system was compiled to function as a standalone application that can be used in any computer environment.

Ibrahim *et al.* [39] introduced a novel approach to classify the risk in DHF patients by using the BIA technique. A total of 184 (97 males and 87 females) serologically confirmed dengue patients (DHF I–IV) were studied during their hospitalization. The relationship between gender and group with the biological electrical tissue conductivity BETC parameters was studied employing univariate analysis of variance. The experimental results showed that BETC, specifically the reactance, was a potentially useful tool in classifying the risk factor of DHF patients.

The work by Ibrahim *et al.* [39] has been extended to diagnose the risk in dengue infections using an artificial neural network technique [57]. The study employed MLFFNN for classifying the risk stages in dengue patients. In this study, the severity of risk in dengue disease was quantified using the dengue patients’ blood data based on threshold values obtained from other researcher’s findings and the WHO classification system [58]. Data comprises of 223 healthy subjects and 207 dengue patients were arranged randomly into the training and testing in the ratio of 70:30. The ANN was trained via the steepest descent back propagation with momentum algorithm method. The optimum network architecture was determined by optimizing the training parameters. The optimization criteria was the sum squared error (SSE) and total classification accuracy of the network. The total classification was subjected to a 25% error tolerance. After the optimal ANN structure was determined, it was pruned using a weight eliminating method to enhance the system performance. The overall classification accuracy was 96.27% with 95.88%, 96.83%, and 95.81% for high risk, low risk, and healthy groups, respectively.

**Figure 4:**
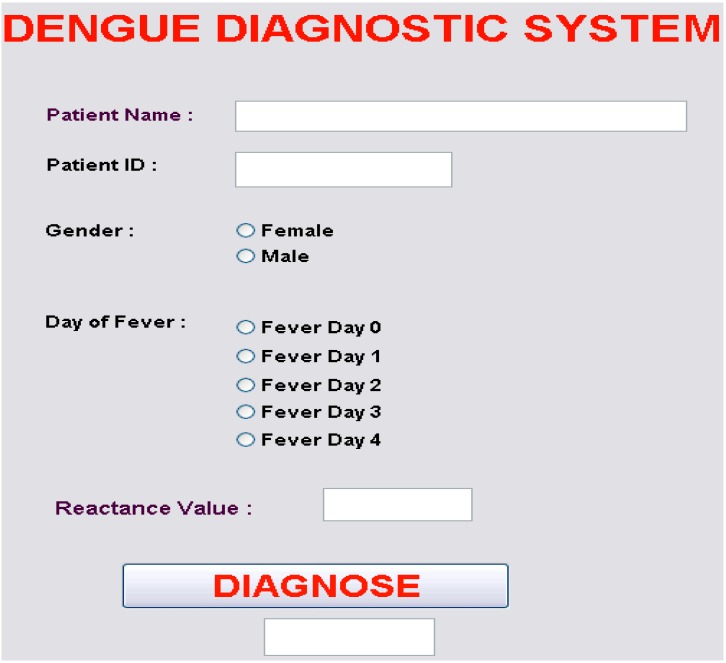
An Automatic dengue risk diagnostic system using artificial neural networks and bioelectric impedance analysis techniques.

Continuing research has taken the neural network architecture obtained from Ibrahim *et al.* [57]. and developed an automatic dengue risk classification. Using the Matlab software, a GUI has been developed for use in real clinical applications as shown in Figure 4. The user interface is comprised of a few dialog boxes and radio buttons that request patient information and input data for the diagnosis process. The DIAGNOSE button is used to initiate MLFFNN calculation for each given input. The system produces one of three outcomes: healthy, low risk, or high risk; and the result is displayed in the bottom most dialog box.

The same technique and the same data were used by Faisal *et al.* [59] to develop a non-invasive intelligent technique for diagnosing the risk stages in dengue patients using clinical manifestations (signs and symptoms) and the BIA measurements. An accuracy of 70% with 0.121 sum squared error was achieved by this model. The study concluded that such a screening system can aid physicians in the diagnosis of the risk and the prognosis of dengue patients, but it will not be definitive.

Although the Multi-Layer Perceptron (MLP) trained via the back propagation (BP) algorithm has demonstrated significant good performance in classification and prediction applications compared to statistical analysis, it suffers from a slow convergence rate and often yields suboptimal solutions [60,61]. To overcome this drawback, many researchers have employed the Levenberg-Marquardt (LM) algorithm [62] or Scaled Conjugate Gradient (SCG) algorithm [36] for training the MLP since these methods provide faster convergence and better estimation results. Those algorithms have been successfully used in medical applications for classifying and diagnosing several diseases.

Faisal *et al.* [59] constructed the dengue patient diagnostic model using the LM and SCG algorithms. Systematic procedures involving training, testing, and validation were followed to construct the diagnostic model so that a higher performance of the diagnostic model can be achieved and the robustness of overall diagnostic models can be maintained. Precise tuning of the internal training algorithms’ parameters was performed to attain the optimal model. Three-layer network is used. The activation function in hidden layer’s neurons is hyperbolic tangent sigmoid while in the output layer’s neurons are sigmoid transfer function. The 5-fold Cross Validation (CV) technique is implemented. The data are divided into five sets; each set contains 101 samples (45 high risk patients, 56 low risk patients). Four sets (404 samples) were used for training and the remaining set was used for testing. The training process was repeated for five times, at each time one of the sets was used as testing set. The results for optimization of the MFNN trained via the Levenberg-Marquardt algorithm showed that the optimal model achieved an average diagnostic accuracy of 70.7% with 73% sensitivity, 74% specificity and a 0.02 average MSE. By implementing the scaled conjugate gradient algorithm, the optimal diagnostic model achieved an average diagnostic accuracy of 75% with 0.01957 average MSE.

##### Adaptive Neuro-Fuzzy Inference System (ANFIS)

Even with the success of ANNs in a decision support system, the use of computerized decision making systems in clinical medicine is rather difficult due to the uncertainty of naturally occurring diseases. In such a situation, fuzzy set theory appears as an appropriate tool for a decision making system since it deals with uncertainty by applying our knowledge and experience directly without any explicit mathematical models. Fuzzy logic describes human thinking and reasoning in a mathematical framework by using several rule bases (IF-THEN) that require a number of human experts to carefully define the rules. Even though the fuzzy logic has been successfully implemented, there are some basic aspects of it that are in need of better understanding. First, the need for a standard method for transforming human knowledge or experience into the rule base and database of a fuzzy inference system is noted. Second, there is a need for effective methods for tuning the membership functions [63]. Based on those needs, the Adaptive Neuro-Fuzzy Inference System (ANFIS) was proposed to serve as a basis for constructing a set of fuzzy if-then rules with appropriate membership functions to generate the stipulated input-output pairs [63].

Faisal *et al.* [64] utilized (ANFIS) to develop a dengue patient diagnostic model. The development of the model was carried out in two steps: defining the initial ANFIS model architecture and training of the defined model. Two approaches were followed to define the initial ANFIS model architecture. In the first approach, the number of membership functions in the inputs and the output were systematically varied and the effect of this variation in the model performance was investigated. In the second approach, a subtractive clustering algorithm was assigned to determine the initial ANFIS model by optimizing the number of membership functions and fuzzy rules. After the initial model structure was defined, it was trained so that the differences between the output obtained from the model and actual output are minimized. The hybrid learning algorithm was employed for this task. The results of the first approach showed that the highest overall accuracy of 80.19% with 71% sensitivity and 86% specificity was achieved. For the second approach, average diagnostic accuracy was 86.13% with 87.5% and 86.7 sensitivity and specificity, respectively.

**Figure 5:**
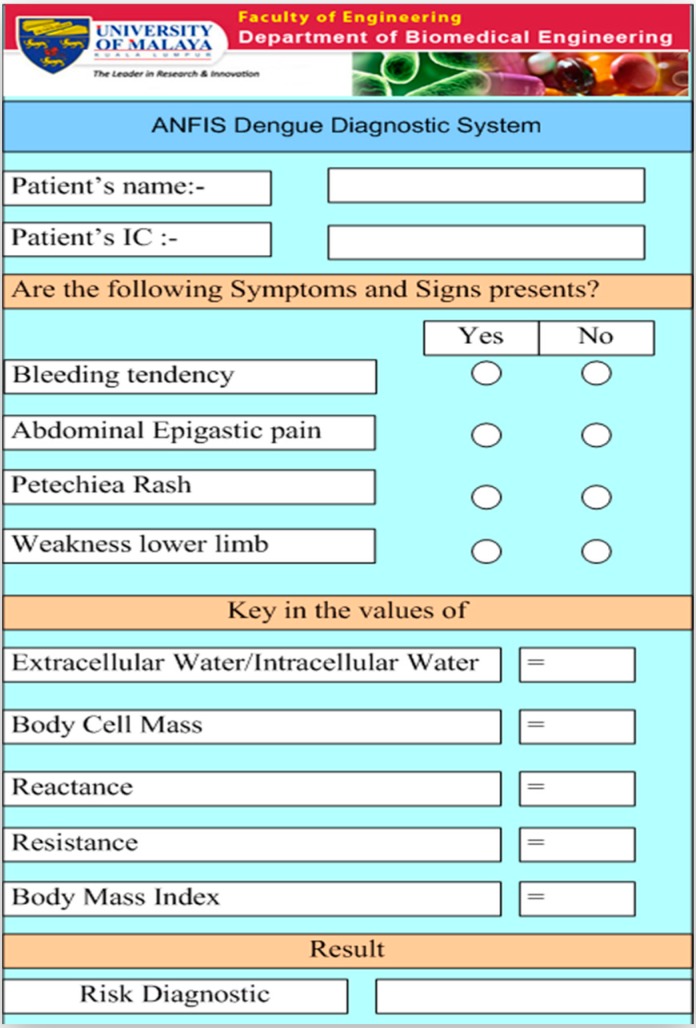
Dengue patient diagnostic system based on Adaptive Neuro-Fuzzy Inference System.

The graphic user interface for the new ANFIS is shown in Figure 5. The user interface is comprised of four parts: patient information such as patient's identification, symptoms and signs, the bioelectrical impedance data, and the dengue patient risk diagnostic which determines the level of risk in dengue patients.

#### 2.1.7. Dengue Fever Detection Using Microfluidic Lab-On-a-Disc (LOD) and Lab-On-a-Chip (LOC) Platforms

Microfluidic devices have been proposed as a solution to overcome the many problems caused by the conventional dengue testing systems such as the high cost, reagent consumption, long turnaround time and complex procedures. Lab-on-a-disc (LOD) and lab-on-a-chip (LOC) are the most popular microfluidic platforms that have been reported by many researchers as a portable diagnostic device for dengue detection [65,66,67].

Ibrahim *et al.* [68] and Yusoff *et al.* [69] have discussed the fundamentals of the microfluidic compact disc (CD) and its application as a platform for ELISA detection of dengue non-structural glycoprotein 1 (NS-1), and Ibrahim *et al.* [68] designed and fabricated a microfluidic CD to automatically perform an ELISA test for dengue detection.

**Figure 6:**
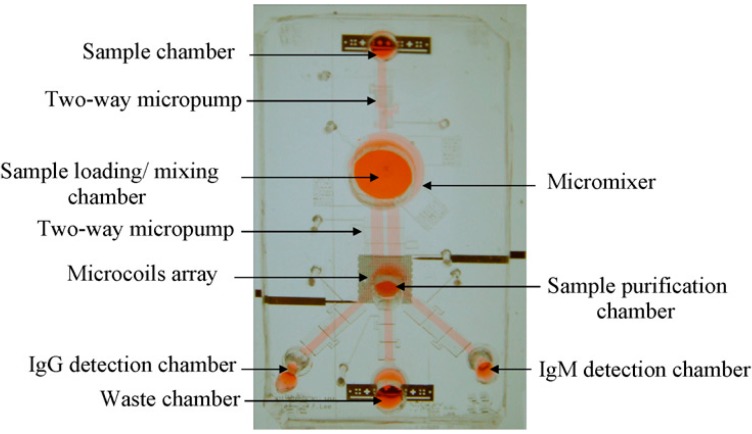
The magnetic bead-based microfluidic chip measures 53 mm × 37 mm. Reproduced with permission [72].

On the other hand, lab-on-a-chip (LOC) has been proposed as a precise, rapid, and low cost platform for dengue detection [70,71]. Lee *et al.* [70] reported an integrated microfluidic platform that can detect the dengue virus by coating magnetic microbeads with antibody. A multi-way micropump shown in Figure 6 is used for moving the serum sample, reagents and other buffers inside the microfluidic net (channels and chambers). Lee *et al.* [72] claimed that the time required to perform one test is 30 min, which is only 1/8th of the time required to detect dengue using a conventional testing platform. Weng*et al.* [71] reported a new microfluidic LOC platform for dengue detection where a suction method (instead of pumping) is used to move the liquid inside the device. Polydimethylsiloxane (PDMS) has been used for the chip fabrication and the surfaces are pre-functionalized to minimized protein adsorption. The dengue testing process can be finished within 30 min and the chip can stay stable for one month if it is stored at 4 °C.

#### 2.1.8. Dengue Virus Detection Using Paper-Based-Diagnostic Platform

The lab-on-paper, or paper-based diagnostic platform is a recently developed low cost method of implementing point of care diagnosis. The paper-based devices are easy to use, low cost, disposable, and can be used for a wide range of biomedical diagnosis [73,74,75].

A medical patch developed by Martinez *et al.* [73] allows for the use of chromatography paper as a low cost, low volume, and portable bioassay. The paper is patterned with test areas which are doped with the required reagents, which change in color when it reacts with the intended analytes. Matthews *et al.* [74] further enhanced the paper-based platform by developing an object identification algorithm that is light enough for use on mobile phones. The application utilizes the built-in camera of mobile phones for image capturing of the medical patch developed by Martinez *et al.* [73]. Once the image is captured, a developed mobile application then performs the necessary image processing and determines the disease state.

In a novel study conducted by Lo *et al.* [75], dengue virus is detected on a paper-based device for the detection of dengue virus by first amplifying the nucleic acids via reverse transcription loop-mediated isothermal amplification (RT-LAMP), then analysing the results using a colorimetric assay on paper. The process of RT-LAMP is performed on Dengue serotype-2 ribonucleic acid (RNA) using conventional microwell assay technique. Once the RT-LAMP process is completed, the DNA product is then dropped on a waxed patterned 96-well paper where it is mixed with biotin-11-deoxyuridine, and then conjugated with streptavidin horseradish peroxidase. The 96-well paper is then washed and scanned to obtain the colorimetric results on a computer.The study concluded that the combination of RT-LAMP andpaper-based colorimetric approach reduces the process time, while requiring very little sample volume and is suitable for the point-of-care application.

### 2.2. Summary

The summaries of biomedical engineering techniques in dengue disease are listed in Table 2, Table 3 and Table 4. Table 2 summarizes the biomedical engineering techniques of ultrasound, echocardiography and electrocardiography (ECG), strain gauge plethysmography, laser Doppler velocimetry and bioelectrical impedance in dengue disease. Table 3 summarizes the biomedical engineering techniques in dengue clinical decision support systems. Table 4 summarizes the biomedical engineering techniques for dengue fever detection using microfluidic lab-on-a-disc (LOD), lab-on-a-chip (LOC), and paper-based platforms.

**Table 2:** Summary of biomedical engineering techniques of ultrasound, echocardiography and electrocardiography (ECG), strain gauge plethysmography, laser Doppler velocimetry and bioelectrical impedance in dengue disease.

Authors	Year	Studies	Methods	Findings/Results
Srikiatkhachorn *et al.* [18]	2007	To delineate the locations and timing of plasma leakage in DHF cases	Ultrasound	The Pleural effusion was the most common ultrasonographic sign of plasma leakage in dengue infection
Venkata *et al.* [19]	2005	Determination of the usefulness of ultrasound in dengue disease severity in patients	Ultrasound	Thickened gall bladder wall, pleural effusion, and ascites should strongly favor the diagnosis of dengue fever
Setiawan *et al.* [20]	1989	Examination of sonographs to identify the relationship between the clinical severity of DHF patients grades I/II and grades III and IV.	Ultrasound	Pleural effusions, ascites and gallbladder wall thickening, pararenal and perirenal fluid collections, hepatomegaly, and pancreatic gland enlargement are detected in in DHF patients grades III and IV
Pelupessy *et al.* [21]	1989	Utilization of echocardiography to diagnose dengue patients	Echocardiography and electrocardiography (ECG)	No signs of pericardial effusion could be determined on physical examination of DHF patients associated with severe shock. However echocardiogram results were able to clearly show a small amount of fluid.
Wali *et al.* [22]	1998	Assessment of cardiac function of DHF and DSS patients	Radionuclide ventriculography, echocardiography, and ECG	Acute reversible cardiac insult may be noticed in DHF/DSS and could be responsible for hypotension/shock seen in some patients.
Yusoff *et al.* [23]	1993	Investigation of echocardiograms and ECGs in healthy and dengue patients	Echocardiography and electrocardiography (ECG)	ECG and echocardiographic abnormalities are common during the acute phase of DHF which can be used as early detection
Gamble *et al.* [24]	2000	Investigation of age-related changes in microvascular permeability in dengue patients	Strain Gauge Plethysmography	Children are more susceptible to develop hypovolaemic shock than adults in DHF
Bethell *et al.* [25]	2001	Assessment of microvascular to differentiate children with DSS, DHF without shock, and healthy children	Strain Gauge Plethysmography	The microvascular permeability for patients with dengue was 50% higher compared to healthy patients. However, there was no significant difference in the permeability between the DSS, DHF dengue patients
Hassan *et al.* [30]	2003	Evaluation of microcirculation changes due to plasma leakage and increase of microvascular permeability in DHF patients	Laser Doppler Velocimetry	The technique can differentiate between normal healthy subjects and DHF patients, but cannot clearly identify the DHF severity stages
Klassen *et al.* [42]	2000	Determining the hydrational profile in dengue patients	Bioelectrical Impedance	Body impedance can be used to monitor the dengue fever progression.
Fang *et al.* [45]	2010	Detection of dengue fever from patient serum	Biosensor	The impedance changes in correlation with concentrations of dengue antibody in serum samples
Ibrahim *et al.* [46]	2004	Modeling of hemoglobin (Hb) status in dengue patients using the BIA parameters	Multiple-linear-regression analysis	Accuracy of 42% was achieved for modeling the Hb variation
Ibrahim *et al.* [46]	2008	Modeling of hemoglobin (Hb) status in dengue patients using BIA parameters	Artificial Neural Network (ANN)	Accuracy of 74% was achieved for modeling the Hb variation

**Table 3:** Summary of biomedical engineering techniques in in dengue clinical decision support systems.

Authors	Year	Studies	Methods	Findings/Results
Ibrahim *et al.* [39]	2005	Classification of dengue patients based on disease severity	Multilayer Feed-Forward Neural Networks	Classification was achieved with accuracy 96.27%, sensitivity 95.88%, specificity 96.83%, and 25% error tolerance
Faisal *et al.* [47]	2008	Distinguish between dengue patients and healthy subjects using self-organizing map	Self-organizing map	4 significant bioelectrical impedance parameters and 3 signs/symptoms were defined to distinguish between the two groups.
Faisal *et al.* [55]	2010	Determination of classification criteria to classify the severity of dengue disease	Self-organizing map	Three criteria were defined to classify the level of risk in dengue patients.
Faisal *et al.* [59]	2010	Classification of dengue patients based on disease severity	MFNN trained via the Levenberg-Marquardt algorithm	Classification was achieved with accuracy 70.7%, sensitivity 67%, specificity 74%
Faisal *et al.* [59]	2010	Classification of dengue patients based on disease severity	MFNN trained via Scaled Conjugate Gradient algorithm	Classification was achieved with accuracy 75%, sensitivity 73%, specificity 74%
Faisal *et al.* [64]	2012	Classification of dengue patients based on disease severity	Adaptive Neuro-Fuzzy Inference System	Classification was achieved with accuracy 80.19%, sensitivity 71%, specificity 86%
Faisal *et al.* [64]	2012	Classification of dengue patients based on disease severity	Adaptive Neuro-Fuzzy Inference System with subtractive clustering algorithm	Classification was achieved with accuracy 86.13%, sensitivity 87.5%, specificity 86.7%

**Table 4:** Summary of biomedical engineering techniques of microfluidic lab-on-a-disc (LOD), lab-on-a-chip (LOC), and paper-based platforms in dengue disease.

Authors	Year	Studies	Methods	Findings/Results
Ibrahim *et al.* [68]	2010	Investigation of the viability of the microfluidic CD as a platform for ELISA	Microfluidic Lab-on-a-Disc	Automated sequencing of ELISA for NS-1 detection can be accomplished on the CD
Yusoff *et al.* [69]	2009	Development of microfluidic CD as a platform for ELISA	Microfluidic Lab-on-a-Disc	Demonstrated a CD design for automated ELISA testing for Dengue NS-1.
Lee *et al.* [70]	2009	Investigation of antibody coated magnetic microbeads for speeding up the ELISA process on the microfluidic CD	Microfluidic Lab-on-a-Chip with Microbeads	Dengue testing time was reduced to 30 min (1/8th of the time compared to conventional 99-microwell method for IgM and IgG ELISA)
Weng *et al.* [71]	2011	Investigation of suction based pumping in microfluidic platform to speed up the ELISA process	Microfluidic Lab-on-a-Chip	Dengue testing time is reduced to 30 min for IgG ELISA test
Martinez *et al.* [73]	2007	Investigation of patterned chromatography paper for Dengue detection	Paper based	Paper based bioassay changes color during detection.
Lo *et al.* [75]	2009	Development of object identification algorithm on mobile phones for analysing the paper based patch developed by Martinez *et al.* [73]	Paper based	Image captured by the mobile application is processed and the disease state is determined
Matthews *et al.* [74]	2007	Development of waxed patterned 96-well paper to perform Dengue virus detection.	Paper based	The 96-well paper allows for colorimetric detection that reduces the process time, while requiring very little sample volume and is suitable for the point-of-care application.

## 3. Malaria

Malaria is an infection that affects humans and some animals and is transmitted by infected female *Anopheles* mosquitoes. Fever, headache, and in some severe cases patients progressing to coma or death, are the common symptoms of this disease. Sub-Saharan Africa, Asia, and the Americas are the areas where malaria is most prevalent [4]. In general, five types of the plasmodium have the capability of causing human infection: *P. falciparum*, *P. vivax*, *P. ovale*, *P. malariae*, and *P. Falciparum* are mainly found in Africa and some parts of Asia and South America [76]. *P. falciparum* is the common cause of severe malaria cases that can lead to death. *P. vivax* is less fatal but can lead to serious anaemia in children. Two hundred sixteen million cases of malaria were reported in 2011, where 81% of those cases were found in Africa [4]. It is estimated that around 86% of these cases are children under 5 years of age. The two common methods for diagnosis of malaria are light microscopy of blood and the rapid diagnostic tests (RDTs) [77]. The main advantage of the light microscopy is the low cost in endemic areas. However, the need for a highly trained operator and the lack of portability are the main drawbacks. RDT testing kits are preferable in other areas but the cost of the RDT kits is high.

### 3.1. Biomedical Engineering Approaches

Several biomedical engineering approaches for malaria detection have been described including microfluidic systems, image processing, and bioelectrical properties of blood. The following sections review these approaches.

#### 3.1.1. Image Processing

Many researchers have reported the auto-detection of malaria infection by utilizing image processing techniques on microscopy images [78,79,80,81,82,83,84,85]. This work is based on images of cultured malarial parasites that were grown under a controlled environment rather than in patients’ blood. Rao [85] utilized the stained images of the *P. falciparum* to analyse its life cycle. At a later study by Sio *et al.* [83], new software that automatically counts the malarial parasite has been reported. The software focused on the counting of *P. falciparum* in images where the regular blood components and other types of noise are not present. The algorithm applied is able to differentiate between infected and uninfected red blood cells, and successfully count the parasites from peripheral blood specimens. Dimension and colour of components were used for identification. Halim *et al* [80] performed template matching techniques to detect red blood cells (RBC). Gray scale images were processed using different image process techniques for parasite detection. The second developed method utilizes the colour co-occurrence array that analyzes pixel colour index and the indicated colour of the surrounding pixels. The various techniques produced results with accuracy of 80%–88% and a sensitivity of 92%–98%.

#### 3.1.2. Microfluidics

The portability and the small volume of sample required to perform a test are the main advantages of the microfluidic platforms [86]. These features, along with the low cost of the microfluidic platform make it ideal to be used on site and in resource-limited areas.

**Figure 7:**
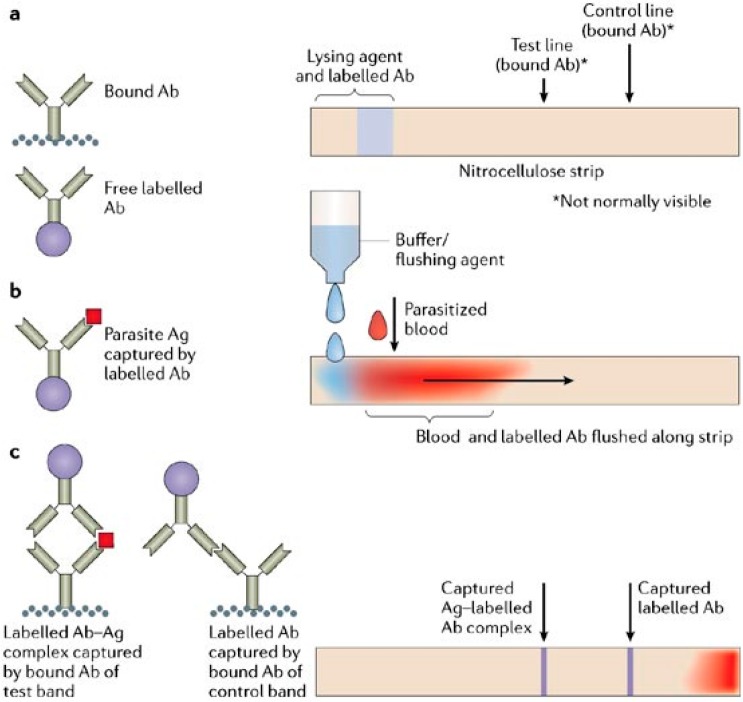
Schematic of the lateral flow strip to diagnose Malaria. (top) Layout of the strip, (middle) Flushing agent is added to help flush parasitized blood along the strip, and (bottom) visible lines indicate presence of antigens in the parasitized blood. Reproduced with permission [89].

Different immunochromatographic methods (dipstick) of malaria infection detection have been described [87,88]. These platforms are easy to use, portable, and can be used in the most challenging environments. Most are based on the detection of malaria antigens from a patient’s blood sample. The targeted antigen types include histidine-rich protein-2 (HRP-2), aldolase, and plasmodium lactate dehydrogenase (pLDH). The developed methods have the ability to differentiate between the main malaria types through the immunogenic differences in the proteins. Figure 7 shows an example of a microfluidic platform (dipstick) that utilizes immunochromatographic lateral flow to detect malarial proteins (antigen (Ag)) that are extracted from patient blood [89]. The disadvantage of the microfluidic platforms is their accuracy which is less than that from gold standard microscopy techniques [90,91]. As the disease severity declines, the concentration of malarial antigens quickly decreases. Therefore, these detection techniques are good for detection of recurrent infection and not for observing the response of the patient to treatment.

Many platforms were proposed using the flow cytometry approaches for malaria detection have been reported [92,93]. Saito-Ito *et al.* [93] presented a fast, high sensitivity, and low error diagnostic platform that is capable of detecting the *P. falciparum* from a sample that contains erythrocytes and stained parasites. The sensing principle was based on forward-angle light scattering from an argon laser, and green fluorescence was utilized in this process. The author claimed that the required time to perform a full test is 2–3 min including sample preparation. Jiménez-Díaz *et al.* [94] proposed a new flow cytometric platform for malaria detection based on observing the differences of infected erythrocytes stained auto-fluorescence and DNA content with that of healthy cells. The author claimed that the proposed platform is rapid, simple to use, sensitive, and can accurately detect malarial pathogens.

#### 3.1.3. Paper-Based Microfluidic Cartridge

A method of paper-based microfluidics has been reported to prepare stained malaria parasites for detection using traditional optical microscopy [95]. Horning *et al.* [95] presented a paper microfluidic cartridge for automated staining of malaria parasites. The cartridge is similar to a dipstick, but replaces the cellulose strip with paper. Blood is dropped onto a piece of dyeing hydrophilic paper which stains the malaria parasites. The blood is then channeled by capillary forces through the paper into an optically transparent slanted chamber which gradually get thinner. The slanted chamber produces a thick smear near the paper, and a thin smear towards the end of the chamber. The cartridge can then be examined using traditional optical microscopy. The authors claimed that the device is easy to use, fast, low cost, has good optical properties, and is suitable for automated microscopy. A comparison with the standard Giemsa smear technique has shown that the cartridge produces smears equal to that of a blood smear as prepared on a microscope slide by an expert microscopist.

#### 3.1.4. Microarray Chip

Recently, Jin *et al.* [96] proposed a microwell array chip that enables the analysis of single live cells. The proposed system is capable to analyze more than 234,000 individual cells rapidly and efficiently.

**Figure 8:**
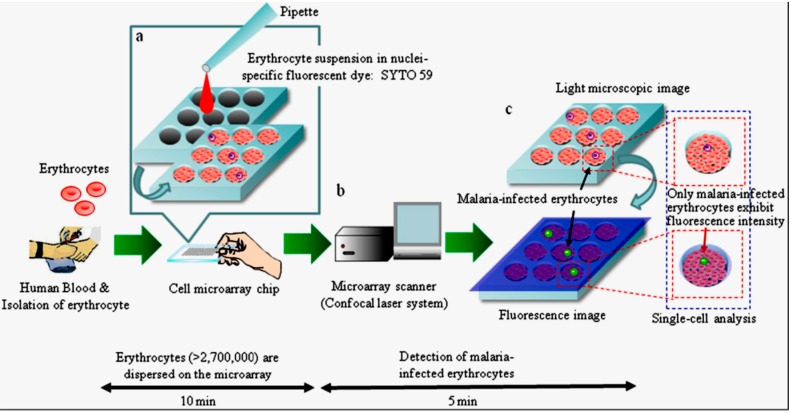
Schematic diagram of the process for detection of malaria-infected erythrocytes on a cell microarray chip. (**a**) Erythrocytes stained with a nuclei-specific fluorescent dye, SYTO 59, for the staining of malaria nuclei dispersed on a cell microarray chip using a pipette, which led to the formation of a monolayer of erythrocytes in the microchambers; (**b**) Malaria-infected erythrocytes were detected using a microarray scanner with a confocal fluorescence laser by monitoring fluorescence-positive erythrocytes; (**c**) The target malaria-infected erythrocytes were analyzed quantitatively at the single-cell level. Reproduced with permission [97]—open access.

Expanding on the work of Jin *et al.* [96], Yatsushiro *et al.* [97] reported a novel high-throughput screening and analysis system for malaria infection using microarray chip with a laser scanner (Figure 8). They utilized a polystyrene chip that contains 20,944 micro-chambers fabricated using molding techniques. The author claimed that the system is 10 to 100 times more sensitive than the conventional light microscopy diagnosis method. This system requires 15 min to detect malaria parasites in erythrocytes extracted from centrifuged blood.

#### 3.1.5. Dielectrophoresis (DEP)

Dielectrophoresis (DEP) technology is utilized by many researchers for cell manipulating and characterization [98]. DEP is the phenomenon that describes the motion of polarizable particles in a non-uniform electric field. It allows for the recognition of differentiated populations of particles based on their relative polarisibility. Separation between the living and dead cells is an example of application of DEP; taking advantage of the unique electrical properties of each bioparticle [99]. Aceti *et al.* [100] found that the dielectric properties of erythrocytes infected with the malarial parasite *P. falciparum* are different from normal erythrocytes. The electrical conductivity of the erythrocyte membrane increases sharply when it is infected with Malaria parasites. This sharp increase was due to changes in the composition, morphology and permeability of the erythrocyte membrane. Therefore, Gascoyne *et al.* [101] utilized the DEP technique to separate the malarial infected cells from the healthy cells by dielectrophoretic manipulation in a non-uniform electric field. Only a few microliters of blood are required to detect malaria on the DEP platform.

#### 3.1.6. Bioelectrical Properties

Lonappan *et al.* [102] studied the microwave characteristics of malaria and normal blood samples using a cavity perturbation technique. This method has many advantages such as its simplicity, rapid performance, reliable results and the small sample volume required to run the test. A significant difference of the conductivity of malaria blood samples when compared with the normal healthy samples was seen at frequencies of 2 to 3 GHz. This measurement is a new novel *in vitro* method of diagnosing malaria at its onset using microwaves, and it will allow precautions to be taken early in the disease course such as proper preventative drugs, which will improve disease prognosis [102].

Wilson *et al.* [103] developed an integrated system with dark-field reflection-mode and cross polarization microscopy for the detection of hemozoin in fresh blood samples. Hemozoin is an iron-containing pigment resulting from the breakdown of haemoglobin, found in the malaria parasite. The presence of hemozoin results in a different light scattering effect when compared to healthy RBCs. The result shows that incorporating both methods doubles the contrast when compared to the individual techniques [103].

### 3.2. Summary

The summary of biomedical engineering techniques in malaria disease is listed in Table 5.

**Table 5:** Summary of biomedical engineering techniques in malaria disease.

Authors	Year	Studies	Methods	Findings/Results
Halim *et al.* [80]	2006	Development of template matching techniques for detection of infection in blood smears.	Image processing	Various image processing techniques presented yield a detection accuracy of 80%–88% with a sensitivity of 92%–98%
Sio *et al.* [83]	2007	Development of algorithm to automatically count malarial parasites in images of filtered blood samples	Image processing	The algorithm is able to differentiate between infected and uninfected red blood cells, and successfully counts the parasites in peripheral blood specimens.
Saito-Ito *et al.* [93]	2001	Development of a fast, high sensitivity, and low error diagnostic platform using light scattering in flow cytometry	Microfluidics—Cytometry	Allows for a full test to be performed in 2–3 min including sample preparation
Jiménez‐Díaz *et al.* [94]	2005	Development of flow cytometric method based on measuring autofluorescence and DNA content in stained infected erythrocytes	Microfluidics—Cytometry	The proposed platform is rapid, simple to use, sensitive, and can accurately detect malarial pathogens.
Horning *et al.* [95]	2014	Development of dipstick-like paper microfluidic cartridge for automated staining of malaria parasites	Microfluidics—Paper-based	The device produces results that are comparable to the standard Giemsa smear technique, and has good optical properties to be examined using traditional optical microscopy.
Yatsushiro *et al.* [97]	2010	Development of high-throughput screening and analysis microarray chip	Microarray chip	The microarray chip containing 20,944 chambers allows for 10 to 100 times higher sensitivity compared to conventional microscopy. It only takes 15 min to detect malaria parasites in erythrocytes extracted from centrifuged blood.
Aceti *et al.* [100]	1990	Investigation of dielectric properties of erythrocytes infected with the malarial parasite	Dielectrophoresis	Electrical conductivity of erythrocyte membrane increases sharply when infected
Gascoyne *et al.* [101]	2002	Investigation of identifying malarial infected cells using dielectrophoretic	Dielectrophoresis	Cell separation allows maria detection using only a few microliters of blood
Lonappan *et al.* [102]	2009	Investigation of microwave characteristics of malaria infected and normal blood samples	Bioelectrical	Using a cavity perturbation technique, significant difference of the conductivity can be detected between malaria infected blood samples and normal healthy samples at frequencies of 2 to 3 GHz.
Wilson *et al.* [103]	2011	Assessment of dark-field reflection-mode and cross polarization microscopy in malaria detection	Bioelectrical	Hemozoin (found in malaria parasite) has a light scattering effect that doubles the contrast in cross polarized microscopy images

## 4. Cholera

Cholera is an acute intestinal infection as a result of ingestion of food contaminated with the *vibrio cholerae* bacteria. This infection has a short period of incubation from two to three days. If treatment is not administered immediately, it causes an enterotoxin that leads to vomiting and excessive flowing diarrhea that results in extensive dehydration. According to the WHO, the incidence of cholera has been growing, with approximately 317,000 cases and 7500 deaths in 2010, demonstrating an increase of 43% in the number of cases and 52% in the number of deaths compared to 2009 [6]. In areas where cholera is endemic, the mortality rate has increased for children and pregnant women. Two types of vaccines are available to protect against this disease, and it is easily treated by intravenous rehydration [104].

Although standard culture methods can be employed for detection of *v. Cholera*, limitations including low accuracy, the requirement of expertise and well equipped laboratory, and lengthy time for diagnosis make it impractical in diagnosis of individual cases. Dark field microscopy and a dipstick assay can be used to achieve a fast identification of *v. Cholerae*. These assays can yield results that differ with the definitive culturing methods, therefore fluorescent monoclonal antibody and PCR-based techniques have been developed to improve sensitivity and specificity of the assays [105].

### 4.1. Biomedical Engineering Approaches

A few biomedical approaches have been investigated to improve the detection of cholera toxin (CT).

#### 4.1.1. Microfluidics

Bunyakul *et al.* [106] developed a microfluidic platform incorporating fluorescence and electrochemical detection techniques for the detection of cholera toxin subunit B (CTB). The microfluidic platform was fabricated using soft lithography on polydimethylsiloxane (PDMS). The sample with CTB concentrations of up to 1000 ng/mL is injected into the platform towards a capture zone withCTB-antibodies and ganglioside GM_1_ receptor immobilized onto magnetic beads. The beads are then immobilized using an external magnet for washing and then transferred to a detection zone for either fluorescence detection, or electrochemical detection. The implementation of the microfluidic platform allows for limits of detection of 6.6 and 1.0 ng·mL^−1^ respectively for the fluorescence and electrochemical techniques.

#### 4.1.2. Impedance Techniques

Due to the low levels of lethal dose (LD) in humans [107], there is a high demand for detection techniques capable to discriminate very low concentrations of the toxin. Labib *et al.* [108] developed an immunosensor assay for the detection of CTB using a capacitive method. The sensor consists of a gold electrode coated with monoclonal antibodies against CTB (anti-CTB) inserted into a capacitive flow cell. Potential pulses of 50 mV at 50 Hz are transmitted through the capacitive flow cell, and the transient currents evoked between the electrode and the capacitive flow cell are recorded and used to calculate the capacitance of the liquid. The capacitance value recorded is found to decrease when CTB binds with the anti-CTB coating on the electrode.

### 4.2. Summary

The summary of biomedical engineering techniques in cholera disease is listed in Table 6.

**Table 6:** Summary of biomedical engineering techniques in cholera disease.

Authors	Year	Studies	Methods	Findings/Results
Bunyakul *et al.* [106]	2009	Investigation of fluorescence and electrochemical detection for CTB on microfluidic platform	Microfluidics	Limits of detection of 6.6 ng/mL and 1.0 ng/mL are achievable respectively using the fluorescence and electrochemical techniques.
Labib *et al.* [108]	2009	Development of capacitive immunosensor assay for CTB detection	Capacitive Impedance Measurement	When a 50 mV 50 Hz signal is applied to the immunosensor (constructed from coated gold electrode in a capactive flow cell), the capacitance of the immunosensor decreases if CTB is present.

## 5. Schistosomiasis

Schistosomiasis is a type of parasitic infectious disease affecting at least 230 million individuals per year in developing countries. It is caused by the trematode parasite worms, of the genus schistosoma. The disease is classified into two major types; (a) intestinal schistosomiasis caused by *S. guineensis*, *S. mekongi*, *S. mansoni*, *S. japonicum*, *and S. intercalatum*, and (b) urogenital schistosomiasis caused by *S. haematobium*. Rapid multiplication and transmission of the schistosoma parasites lead to more infected people each year. Furthermore, there is a high mortality rate of this disease, above 200,000 deaths per year in sub-Saharan Africa [5]. Shistosomiasis infection happens when parasites in larval forms produced by snails in fresh water, penetrate the skin, often of the feet, that is exposed to infested water. The larva then turns into mature schistosomes in the body. The adult female worms can produce eggs in the blood vessels causing immune reactions and damaging tissues while others get out of the body through urine or faeces.

In women, urogenital schistosomiasis can be considered as one of the risk factors for being infected by human immunodeficiency virus (HIV). The body does not react to the worm but to the worm’s eggs. Intestinal schistosomiasis can cause abdominal pain, diahrrea, enlargement of the liver, blood in the stool, fluid accumulation in the peritoneal cavity, abdominal hypertension, and spleen enlargement. Urogenital schistosomiasis, on the other hand, can cause haematuria, bladder and ureter fibrosis, damage to kidney, and bladder cancer (as a possible late-stage complication).

Microscopic identification of eggs in specimens of stool or urine is the most efficient technique for Schistosomiasis diagnosis. For detection of shistosomiamansoni and shistosomiajaponicum, only microbiological methods such as the Kato-Katz technique can be applied. The severity of infection rate is determined based on the number eggs per gram of stool sample [109]. However, this diagnostic test is time-consuming, costly, and requires the skill of a trained parasitologist; all of which limit its utility in the developing world.

### 5.1. Biomedical Engineering Approaches

The following sections demonstrate the most common biomedical engineering approaches for diagnosis and prognosis of schistosomiasis including: bioelectrical impedance analysis (BIA), ultrasound, computerized tomography (CT), and magnetic resonance imaging (MRI). Imaging techniques are considered as one of the most rapid, reliable, and accurate methods for detection of schistosomiasis.

#### 5.1.1. Bioelectric Impedance Analysis (BIA)

BIA is an efficient method to assess the total body water (TBW) and its distribution between ECW (Extracellular Water) and ICW (Intracellular Water), and it has been shown that BIA is sensitive to TBW percentage alterations and the ECW to ICW ratio [110] that can provide an easy, inexpensive analysis of body composition. In De Lorenzo *et al.*’s [110] study, body hydration, being defined as total body water per kg of body weight, is considerably higher in patients with schistosomiasis than in normal controls. De Lorenzo *et al.* [110] recruited schistosomiasis patients without clinical symptoms such as no visible fluid retention, no cardiac or renal abnormalities from the underlying disease. BIA was used to measure the amount of TBW and it was found that the percentage of body water is higher in patients with schistosomiasis than in controls (62.9% ± 3.6% *vs.* 57.4% ± 4.3%, *p* < 0.0005). Although, these patients’ anthropometric characteristic was similar with the control group, their TBW% was higher. De Lorenzo *et al.* [110] discusses that various reasons may have partially contributed the increased subclinical alterations in body hydration in patients with schistosomiasis. For example, the subclinical alterations may be due to the arm fat area of schistosomiasis patients being reduced to 20% lower than the control group [110]. However, it is observed that difference in TBW% is small and may not be sufficient to allow a classification.

Hypoalbuminaemia is a cause of oedema. In the absence of fluid retention, albumin levels <1.5–2 g/dL are required to have a clinically detectable increase of in ECW relative to TBW (ECW%) [111]. In De Lorenzo *et al.*’s [110] findings, one of the schistosomiasis patients experienced a 1.9 g/dL and higher level with the absence of oedema. The findings suggest that albumin is vital in controlling body water homeostatis in conditions of normal and subclinically altered body hydration. The findings concluded that schistosomiasis subjects show an apparent subclinical increase in body hydration which is related to the prediction of TBW from BIA.

#### 5.1.2. Ultrasound Imaging of Hepatosplenic-Schistosomiasis

**Figure 9:**
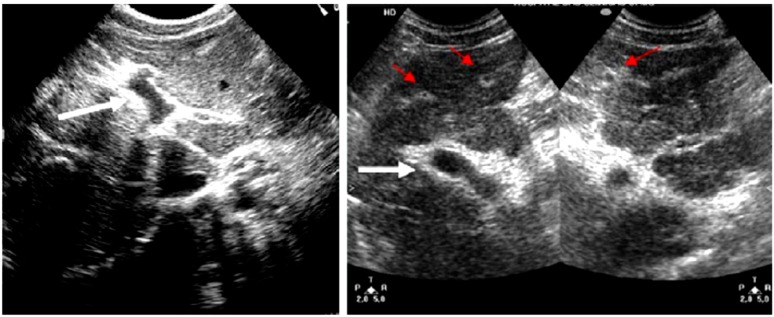
Ultrasound B-scans of the abdomen showing changes caused by schistosomiasis. White arrow: Central periportal fibrosis, red arrows: fibrosis on the periphery of the liver in a patient diagnosed with advanced hepatosplenic schistosomiasis Reproduced with permission [112].

Ultrasound (US) is known to be one of the most effective methods for investigating liver and spleen abnormalities caused by schistosomamansoni. Portal hypertension induced by the periportal fibrosis is shown to be one of the main characteristics of the hepatosplenic form of schistosomiasis, it can be demonstrated by ultrasound imaging through measurement of the diameter of the portal vein and its main tributaries (splenic vein and superior mesenteric vein). Hepatosplenic schistosomiasis can induce formation of collateral vessels in the gastrointestinal tract leading to acute bleeding or possibly death. This phenomenon can be demonstrated by ultrasound imaging indicating an increased risk of bleeding (Figure 9). Ultrasound can also be applied as a diagnostic tool in the assessment of treatment efficacy and regression of fibrosis in schistosomiasis [112,113].

#### 5.1.3. Computerized Tomography (CT) and Magnetic Resonance Imaging (MRI)

Diagnosis of hepatosplenic Schistomiasis can be obtained by observing characteristic changes of the attenuation in the computerized tomography (CT) or signal intensity and enhancement pattern of the magnetic resonance images (MRI) [112,114]. The CT and MRI findings are usually compared and are in agreement with the ultrasound findings. In CT, low-density periportal zones are demonstrated as periportal thickening when compared with echogenic layers in ultrasound images. These low-intensity zones extend uniformly throughout the liver lobes and are enhanced after an intravenous injection of contrast agents to become either homogenous with or denser than the surrounding hepatic tissue.

**Figure 10:**
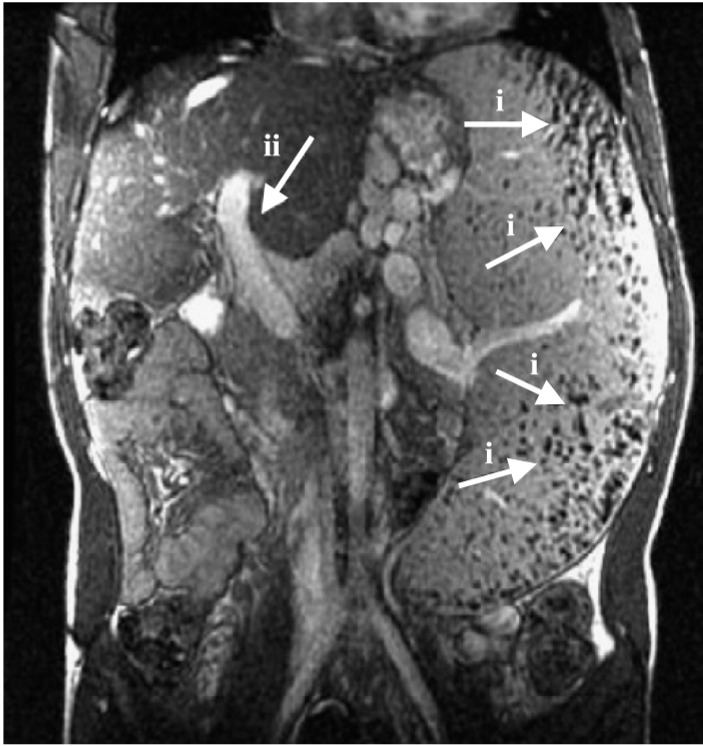
MRI: Gamma-Gandy bodies (siderotic nodules) pointed by arrows labelled “i” in the spleen of a patient diagnosed with hepatosplenicschistosomiasis. Arrow labelled “ii” shows the portal vein. Reproduced with permission [112].

In MRI, on the other hand, periportal bands are demonstrated as areas of hyperintensity on T1-weighted sequences and hyperintensity on T2-weighted sequences after the intravenous injection. Studies of acute cases applying MRI demonstrated that regions of periportal fibrosis were hyperintense on T2-weighted images in all patients (Figure 10). MRI has been known as a highly responsive imaging technique for many diffused liver pathologies. Unlike ultrasound imaging, MRI is not a dynamic method and also it is not dependent on the examiner.

In Japan, ultrasound, CT and MRI techniques and endoscopic examinations with biopsies have been used to detect lesions related to schistosmiases japonica [114]. Portal hypertension is detected by CT, Ultrsound and gastroscopic examination. The MRI imaging diagnoses were performed on chronic lesions and/or sequelae of schistosemiasis and in well-equipped hospitals because of its high cost and longer time of examination.

### 5.2. Summary

The summary of biomedical engineering techniques in schistosomiasis disease is listed in Table 7.

**Table 7:** Summary of the biomedical engineering techniques in schistosomiasis disease.

Authors	Year	Studies	Methods	Findings/Results
De Lorenzo *et al.* [110]	1997	Measurement of body water using BIA in schistosomiasis patients	Bioelectric Impedance Analysis (BIA)	Percentage of body water is higher in patients with schistosomiasis than in controls (62.9% ± 3.6% *vs.* 57.4% ± 4.3%, *p* < 0.0005).
Lambertucci *et al.* [112]	2008	Detection of liver and spleen abnormalities caused by shistosomamansoni using ultrasound	Ultrasound imaging	Symptoms of schistosomiasis (such as liver and spleen abnormalities, formation of collateral vessels in the gastrointestinal tract, *etc.*) can be detected using ultrasound imaging
Cheung *et al.* [113]	1996			
Lambertucci *et al.* [112]	2008	Application of CT and MRI in detecting schistomiasis symptons	Computerized Tomography (CT) and Magnetic Resonance Imaging (MRI)	In CT, low-density periportal zones can be seen as periportal thickening when compared with echogenic layers in ultrasound images. In MRI, periportal bands can be seen as areas of hyperintensity on T1- and T2-weighted sequences.
Ohmae *et al.* [114]	2003	Application of Ultrasound, CT and MRI in detecting schistomiasis symptons	Ultrasound, Computerized Tomography (CT), Magnetic Resonance Imaging (MRI)	Portal hypertension can be detected by CT and US. MRI imaging is more suitable for chronic lesions and/or sequelae of schistosemiasis

## 6. Lymphatic Filariasis

Lymphatic Filariasis which is also known as elephantiasis, is responsible for more than 40 million disfigured and infirm people, and intimidating more than 1.3 billion people in 72 countries [8]. The disease starts when an infected mosquito bites a human and transmits filarial parasites to the human lymphatic vessels. Then, three types of filarial worms develop inside the infected body: *Wuchereria bancrofti*, *Brugia malayi* and *Brugia timori*. These worms settle in the lymphatic system for 6–8 years. During this incubation period, they produce large numbers of microlariae (larvae) that interfere with the immune system. Although infection typically takes place during the childhood period, painful disfiguration will occur later in life [8].

There are two main approaches for diagnosing lymphatic filariasis: microscopic examination and serological tests. The first technique is to examine blood smears under the microscope to detect microfilariaes. The blood must be collected at night; since the microfilariaes circulate in blood only at night. Although this technique has a low sensitivity, it is still considered as an acceptable alternative diagnostic method when antigen testing cannot be done [115]. On the other hand, serological tests are based on the fact that filarial infection increases the concentrations of antifilarial IgG and IgE in the blood. Thus, one can identify antifilarial immunoglobulin type G and E antibodies (IgG, IgE) or filarial antigens using ELISA which leads to a higher sensitivity [115].

### 6.1. Biomedical Engineering Approaches

Few techniques have been investigated in order to overcome the limitations of lymphatic filariasis diagnosis. The following sections show the available technologies of ultrasonography, lymphoscintigraphy and immunochromatography.

#### 6.1.1. Ultrasonography

Ultrasonography has been used to locate and study the movements of active adult filarial worms of *Wuchereria bancrofti* in the scrotal region in men as a diagnostic tool for lymphatic filariasis; while only a few cases of adult filariae are observed by ultrasonography in women. This movement of *Wuchereria bancrofti* is described as “filaria dance”; which is considered a sign of the presence of *Wuchereria bancrofti* worms [116].

Dreyer *et al.* [117] used longitudinal ultrasound examination to investigate the microfilaria dance sign in the scrotal lymphatic vessels. They found out that ultrasound is a beneficial tool to evaluate in a direct and fast way the usefulness of antifilarial drugs. In another study done by Mand *et al.* [116], they used a portable ultrasound unit, a 7.5 MHz linear transducer and a 3.5 MHz curved array transducer to examine different positions in subjects’ bodies; in order to locate worm nests. The findings showed that ultrasonography is suitable for diagnosis of *Wuchereria bancrofti* infection in female patients as well as males.

#### 6.1.2. Lymphoscintigraphy

Radioisotope imaging (scintigraphy) was demonstrated by Freedman *et al.* [118] as an effective way to evaluate severity of the lymphatic filariasis; however, the primary diagnosis must be done by non-imaging laboratory assays.

#### 6.1.3. Immunochromatography

Weil *et al.* [119] described an immunochromatographic filarial antigen test using specific monoclonal and polyclonal antibodies. The test is done by adding the serum onto a pink sample pad which contains dried polyclonal antifilarial antibodies attached to colloidal gold. Then two drops of a wash buffer are added to a separate wash pad. If the serum is infected, the labelled antibody and filarial antigen move to the top of the card through a nitrocellulose strip resulting in a visible pink line. This technique does not need professional operators; since its easy procedure can be done by individuals without much training. Moreover, the results are accessible within only few minutes, making this test a potentially fast diagnostic tool for lymphatic filariasis infection.

### 6.2. Summary

The summary of biomedical engineering techniques in lymphatic filariasis disease is listed in Table 8.

**Table 8:** Summary of biomedical engineering techniques in lymphatic filariasis disease.

Authors	Year	Studies	Methods	Findings/Results
Dreyer *et al.* [117]	1995	Application of longitudinal ultrasound in detecting lymphatic filariasis infection	Ultrasonography	The microfilaria dance sign in the scrotal lymphatic vessels can be detected using ultrasonography.
Mand * et al.* [116]	2004	Detection of lymphatic filariasis infection using a 7.5 MHz linear transducer and a 3.5 MHz curved array transducer	Ultrasonography	Using ultrasonography, infestation of *Wuchereria bancrofti* can be detected by examining different positions in both male and female subjects’ bodies
Freedman * et al.* [118]	1994	Application of radioisotope imaging (scintigraphy) in detecting lymphatic filariasis infection	Lymphoscintigraphy	Radioisotope imaging allows the evaluation of the severity of the lymphatic filariasis infection
Weil * et al.* [119]	1997	Development of an antigen test using sample pad containing dried polyclonal antifilarial antibodies attached to colloidal gold	Immuno-chromatography	The sample pad produced a visible pink line when infected serum is added to it. The technique does not need professional and produces results within a few minutes.

## 7. Ebola

The first recognition of Ebola virus infection occurred in 1976 in Zaire, Africa and the mortality rate was 88% [7]. There are four types of Ebola virus including: Zaire, Sudan, Ivory Coast, and Reston; the first two being responsible for the majority of hemorrhagic human fever resulting in the high mortality rate [120]. Ebola can be confused with other diseases such as malaria, since the most frequent symptoms include: fever, weakness, vomiting, diarrhea, sore throat and general aches [121]. The formal laboratory diagnostic technique is an ELISA, but this technique is not sensitive enough for all different stages of disease and is not easily accessible in countries endemic for Ebola infection [122].

### 7.1. Biomedical Engineering Approaches

Although, ELISA is the routine test for Ebola, two techniques, RT-PCR (reverse transcriptase polymerase chain reaction) and an optical biosensor have led to high sensitivity and specificity.

#### Optical Immunosensor

An optical immunosensor based on the photo immobilization technique to detect the two most common types of Ebola virus, Zaire and Sudan was developed by Petrosova *et al.* [120]. The sensor was fabricated by coating the tip of a fiber optic with a 200 nm layer of indium tin oxide (ITO), and later with polypyrrole benzophenone. The tip is then irradiated with a 345 nm wavelength light at intensity of 80 mW·cm^−2^ to excite the benzophenone radicals to bind to the Ebola virus antigen. The fiber tip then goes through a process similar to a standard ELISA. The detection is then performed via chemiluminescence measurements using a photo multiplier tube sensor. The results show that the immunosensor is able to detect titers as low as 1:960,000 and 1:1,000,000 respectively for the Zaire and Sudan strains.

### 7.2. Summary

The summary of the biomedical engineering technique in Ebola disease is listed in Table 9.

**Table 9:** Summary of the biomedical engineering technique in Ebola disease.

Authors	Year	Studies	Methods	Findings/Results
Petrosova *et al.* [120]	2007	Development of immunosensor for Zaire and Sudan trains using fiber optic	Optical Immunosensor	The immunesensor is able to detect Zaire and Sudan strains in titers as low as 1:960,000 and 1:1,000,000 respectively

## 8. Leprosy

Leprosy is a chronic infectious disease caused by Mycobacterium leprae. The cause of the disease was identified in 1873 by G.H.A. Hansen and is also known as Hansen’s disease [123]. It remains a public health problem and transmission continues, although prevalence has been reduced in the past half century. Never the less leprosy remains as a leading infectious disease causing disabilities. At the beginning of 2011, the global registered prevalence of leprosy from 130 territories stood around 192,000 cases, while during 2010 the number of newly detected cases was about 228,000 [9]. The primary external sign of leprosy is skin lesions that if left untreated, can be progressive and cause permanent damage to the limbs, eyes, nerves and skin. Thus early diagnosis and treatment is essential [124].

Mycobacterium leprae cannot be cultured in the laboratory, hence scientists are using innovative techniques to measure the interaction of host cells with Mycobacterium leprae, however there still is no primary method for prevention of leprosy nor a means of diagnostic and prognostic testing that is practical in routine clinical care [125]. Problems with diagnosis of leprosy are related to its insidious nature with sometimes conflicting immunological, clinical and pathological manifestations even though there is minimal genetic variation among Mycobacterium leprae isolates worldwide.

An effective diagnosis method for all stages of the diseases is electroneuromyography (ENMG). ENMG is a technique where the muscle nerve under study is stimulated with electric current. The effectiveness is demonstrated by the presence of electroneuromyographic alterations among 98% of leprosy confirmed patients, especially in the diagnosis of pure neural leprosy [126].

### 8.1. Biomedical Engineering Approaches

Although highly sensitive and specific diagnostic tests for Mycobacterium leprae have yet to be established. Imaging techniques and biosensors, especially those based on employing DNA based markers, are considered as strong promising leprosy diagnostic techniques.

#### Diagnostic Imaging

The incidence of bone lesions in leprosy is low, but the radiologic findings of chronic and acute osteomyelitis that are similar to lesions of other granulomatous infectious agents are seen. Employing imaging techniques various degrees of reabsorption of the body associated with leprosy involving feet and hands with loss of disorganizing arthropathies and digits in small joints can be visualized [126,127].

Magnetic resonance imaging (MRI) and ultrasonography (US) have been used as imaging techniques for evaluation of pure neural leprosy. These imaging techniques employ specific markers and they have been used to assist in determining diagnosis and prognosis. US provides high resolution images of the morphological alternations in peripheral nerves; however the value of this technique in the diagnosis of peripheral neuropathy is poorly understood. Moreover, the application of MRI in leprosy peripheral nerve involvement has been described in the literature, and there have been studies of leprosy diagnosis using these techniques [127]. Goulart & Goulart [126] have shown that MRI and Doppler ultrasonography have a sensitivity of 92% and 74%, respectively, to identify active reversal reactions, based on the observation of the color flow signals. However, in case of tender neuropathy, MRI may exclude nerve abscess, while US examination can be done more rapid and effective than MRI imaging.

### 8.2. Summary

The summary of the biomedical engineering technique in leprosy disease is listed in Table 10.

**Table 10:** Summary of the biomedical engineering technique in leprosy disease.

Authors	Year	Studies	Methods	Findings/Results
Goulart & Goulart [126]	2008	Applications of MRI and US in detecting symptoms of Leprosy	Magnetic resonance imaging (MRI) and ultrasonography	MRI and ultrasonography have a sensitivity of 92% and 74% respectively in identifying active reversal reactions. Ultrasonography examinations can be done more rapidly and effectively than MRI imaging.

## 9. Leishmaniasis

Leishmaniasis is a neglected parasitic disease that is caused by the protozoan parasite of the Leishmania genus and spread by the bite of female phlebotomine sand flies [12,128]. These flies inject the infective stage of promastigote parasites into human hosts [10]. These are then phagocytized by macrophages and transformed into amastigotes which will develop in the infected cells and cause skin lesions that will take months or even years to heal [10] or which if left untreated, may be fatal [128]. Leishmaniasis is a public health problem that has being reported to have a 500% increasing incidence in the most endemic areas in Africa, Asia, the Middle East, and the Mediterranean [10,11].

The established gold standards for Leishmaniasis detection involve the isolation of parasites either microscopically or by culture [12]. Existing available diagnostic tests include histology, culture, molecular techniques, Leishmanin skin test and serologic skin tests. Never the less those diagnostic tests have their own limitations particularly due to false positives with Chagas disease and also to a lesser extent to tuberculosis and leprosy [10,12].

### 9.1. Biomedical Engineering Approaches

To overcome the limitations of current diagnostic techniques, integrated electronic biosensors systems that are suitable for field diagnosis are being developed [12].

#### Impedance-Based Biosensor

Perinoto *et al.* [12] developed a nanostructured biosensor system to detect specific anti-leishmania antibodies by measuring the capacitance of proteoliposomes integrated onto gold interdigitated electrodes. These electrodes were functionalized by attaching antigenic proteins to the surface through repeated immersing of the electrodes into a solution containing the antigenic proteins. Electrical capacitance measurements of this structure containing immobilized proteoliposomes allow the recognition of specific anti-Leishmania amazonensis antibodies at the concentration of 10^−5^ mg/mL [12]. This technique can be applied for the diagnosis of other protozoan and bacterial infectious diseases such as the tuberculosis, malaria, filariasis, schistosomiasis, onchocerciasis and other neglected diseases.

### 9.2. Summary

The summary of the biomedical engineering technique in Leishmaniasis disease is listed in Table 11.

**Table 11:** Summary of the biomedical engineering technique in Leishmaniasis disease.

Authors	Year	Studies	Methods	Findings/Results
Perinoto *et al.* [12]	2010	Development of nanostructured biosensor system by integrating proteoliposomes onto gold interdigitated electrodes.	Impedance-based biosensor	Measuring the electrical capacitance of the biosensor allow the recognition of specific anti-Leishmania amazonensis antibodies at concentration of 10^−5^ mg/mL

## 10. American Trypanosomiasis (Chagas Disease)

American trypanosomiasis, or Chagas disease is endemic in tropical areas and is caused by the parasite Trypanosome cruzi. The parasite is transmitted by the triatomine family of bugs (commonly known as “kissing bugs”) that act as reservoirs and vectors in endemic areas. As of 2002, 5–6 million people were infected worldwide, with 25 million more at risk [129]. The Disease is typically found in Latin America, however, due to population mobility, the disease has been increasingly detected in North America, Europe and Western Pacific countries [129].

There are two phases of the disease: acute and chronic. The acute phase starts when the parasite Trypanosome cruzi enters the body, and may last from 4 to 8 weeks. Local reaction at the point of entry such as skin irritation occurs due to the bug bite or entry through the conjunctiva. Once Trypanosome cruzi enters the body, the patient may experience common symptoms such as fever, vomiting, diarrhoea, and anorexia. During this phase, a patient experiences acute myocarditis, where an ECG may show signs of sinus tachycardia, first degree atrio-ventricular (A-V) block, low QRS voltage, or primary T-wave changes. The chronic phase starts when the parasite can no longer be detected in the blood, and symptoms of myocarditis and meningoencephalitis disappear. In this phase, detection can only be done through serological tests such as IgG antibody detection. In the chronic phase, 10%–30% of patients may suffer cardiac damage, while others may experience damage to the cardiac conduction network and the autonomic nervous system [129,130].

For diagnosis of the acute phase of infection, parasites can be detected by direct parasitological tests. Thin and thick blood smears allow microscopic observation of the parasite. [129,130]. In the chronic phase, only 50% of the patients may show positive results using parasitological tests. Serological tests such as haemagglutination, indirect immuno fluorescence, and ELISA are more applicable. Current approaches of detection have limitations in terms of consistency of results, and requiring at least two techniques to be positive in order to properly diagnose Chagas infection. However, as untreated Chagas may lead to damage to the cardiac conduction and autonomic nervous system, biomedical engineering approaches have been investigated and developed for a more accurate diagnosis of the disease.

### 10.1. Biomedical Engineering Approaches

In order to overcome the difficulties faced in Chagas diagnosis, biomedical engineers have developed more accurate tools to help diagnose and identify the disease. This instrumentation consists of biosensors that are based on electric impedance and current measurements.

#### Biosensors

A Chagas disease biosensor has been reported by Diniz *et al.* [131]. It had gold and platinum electrodes that were coated with an oxide layer and recombinant antigens (cytoplasmic repetitive antigen and flagellar repetitive antigen for Chagas), and dipped in patient’s serum. The study showed that complex impedance increases much faster when the electrodes are dipped in Chagas positive serum, compared to a slower increase in impedance when dipped in Chagas negative serum.

In a similar study by Ribone *et al.* [132], a biosensor utilizing gold electrodes was constructed. Similar to the Chagas ELISA method, the electrode is covered with Trypanosome cruzi antigen that reacts with patients’ anti-Trypanosome cruzi IgG. A classic enzyme label using horseradish peroxidase conjugated with anti-human IgG was applied. However, instead of using the typical optical detection method, a potential of less than 0.1 V was applied and electrode current was measured. An increase in current indicates an increase in IgG antibody. In a separate study by Belluzo *et al.* [133], the same biosensor was improvised with custom made chimeric receptors that react with the IgG antibody. The biosensor was shown to achieve 100% specificity while attaining increased sensitivity with a detection limit that is eight times lower compared to commercially available ELISA kits.

### 10.2. Summary

The summary of biomedical engineering techniques in Chagas disease is listed in Table 12.

**Table 12:** Summary of biomedical engineering techniques in Chagas disease.

Authors	Year	Studies	Methods	Findings/Results
Diniz *et al.* [131]	2003	Development of biosensor using gold and platinum electrodes coated with an oxide layer and recombinant antigens	Biosensors	Complex impedance of biosensor increases much faster when dipped in Chagas positive serum (as compared to Chagas negative serum)
Ribone *et al.* [132]	2006	Development of biosensor using gold electrodes covered with Trypanosome cruzi antigen.	Biosensors	When applied to samples containing anti-Trypanosome cruzi IgG, an increase in current is measured.
Belluzo *et al*. [133]	2011	Enhancement of biosensor by Ribone *et al.* [132] using chimeric receptors	Biosensors	The enhancement produced 100% specificity and increased sensitivity. The detection limit is eight times lower than commercial ELISA kits

## 11. Conclusions and Outlook

This paper has reviewed the biomedical engineering approaches for diagnosing and monitoring tropical diseases. Methods such as ultrasound, echocardiography and electrocardiography, strain gauge plethysmography, laser Doppler and bioelectrical impedance and other techniques allow a noninvasive approach to tropical disease diagnosis and management while overcoming the limitations of conventional approaches. Clinical decision support systems based on self-organizing maps, multilayer feed-forward neural networks and adaptive neuro-fuzzy inference systems assist clinicians to obtain a more specific diagnosis of the disease type and stage, whereas lab-on-chip and micro/nanofluidics and photonic approaches allow for a more sensitive and specific diagnosis at lower cost and a shorter turnaround time. Although there has been much progress, there are still many opportunities to improve these current biomedical engineering approaches as well as to develop new approaches for the described and other tropical diseases.
